# A Novel Mutation in ATRX Causes Alpha-Thalassemia X-Linked Intellectual Disability Syndrome in a Han Chinese Family

**DOI:** 10.3389/fped.2021.811812

**Published:** 2022-01-20

**Authors:** Shaomin Wu, Yingchun Zheng, Cailing Xu, Jiahui Fu, Fu Xiong, Fang Yang

**Affiliations:** ^1^Department of Fetal Medicine and Prenatal Diagnosis, Zhujiang Hospital, Southern Medical University, Guangzhou, China; ^2^Prenatal Diagnosis Center, Affiliated Dongguan Hospital, Southern Medical University (Dongguan People's Hospital), Dongguan, China; ^3^Department of Medical Genetics, School of Basic Medical Sciences, Southern Medical University, Guangzhou, China

**Keywords:** ATRX, α-thalassemia, intellectual disability, novel mutation, whole-exome sequencing

## Abstract

**Objective:**

To analyze genetic mutations in a Chinese pedigree affected with Alpha-thalassemia X-linked intellectual disability syndrome, providing a precise diagnosis and genetic counseling.

**Methods:**

Clinical data was collected. A novel alternative splicing variant detected by whole-exome sequencing was validated by Sanger sequencing. The functional effect of the mutation was predicted with Mutation Tasting. The analysis of 5′ splice site score was estimated with MaxEntScan. Changes in amino acid sequencing were predicted with Mutalyzer. The tertiary structures of the wild type and mutation-carrying protein were predicted by I-TASSER. RNA was extracted from peripheral blood lymphocytes from the proband, his mother and a healthy control. Quantitative Real-Time PCR was used to detect mRNA expression.

**Results:**

The proband presented with severe intellectual disability, developmental delay, characteristic facies, seizures and cryptorchidism. A novel hemizygous duplication mutation in the *ATRX* gene in a splice site between exons 3 and 4, NM_000489: c.189+1dupG, was identified with WES in the proband. Sanger sequencing confirmed that the mutation was inherited from his mother, who carried a heterozygous mutation, while his father was not affected. Bioinformatics analysis indicated that the splicing region where the mutation was located is highly conserved and the variant was damaging, producing a truncated protein due to the premature translation of a stop codon. Sanger sequencing with the Quantitative Real-Time PCR product containing a G base inserted between bases 189 and 190. The level of mRNA expression showed that *ATRX* gene transcription decreased due to the mutation (*P* < 0.05).

**Conclusions:**

A novel mutation in *ATRX* was found in this pedigree and was confirmed to be pathogenic through functional studies. Our research expanded the spectrum of *ATRX* gene mutations, providing a precise diagnosis and a basis for genetic counseling.

## Background

Alpha-thalassemia X-linked intellectual disability syndrome (ATR-X syndrome) is a rare X-linked dominant disorder characterized by intellectual disability, developmental delay, characteristic facies, seizures, gastrointestinal dysfunction and genital anomalies. Additionally, some patients have α-thalassemia (1). According to different mutations, affected individuals suffer from mild to severe intellectual disability, typically in the severe-to-profound range. All milestones are delayed. The majority of the patients are unable to speak, although some can use a few words or signs (2). Distinctive facial traits include microcephaly, upswept frontal hair, a flat nasal bridge, an open mouth and excess salivation (3). ATR-X syndrome is caused by mutations in the *ATRX* gene, which is located on Xq21.1, containing 37 exons and spanning approximately 280 kb. The encoded ATRX protein contains an ATPase/helicase domain, belonging to the SWI/SNF family of chromatin remodeling proteins. It participating in a wide range of biological functions: transcriptional regulation, DNA repair and chromosome segregation (4). The latest researches show that the mutant *ATRX* gene is associated with an increased risk of osteosarcoma and lower-grade gliomas (5, 6). One hundred and twenty-seven disease-causing mutations have been reported, including deletions, insertions, non-sense mutations and splicing variants (4, 7). Here, we investigated a Han Chinese family with ATR-X syndrome and detected a novel alternative splicing variant through whole-exome sequencing (WES). We studied the function and potential pathogenic mechanism of the mutant gene and found that the novel mutation resulted in haploinsufficiency of the *ATRX* gene, which caused ATR-X syndrome.

## Methods

### Case Presentation

**Figure 1:**
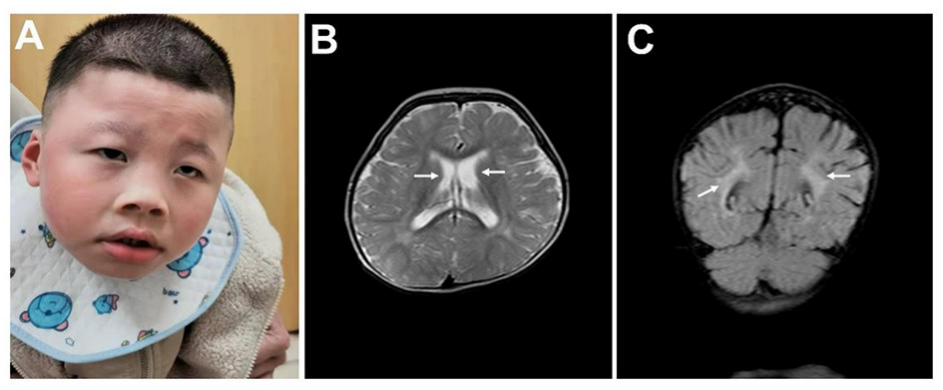
Clinical data. **(A)** Typical facial appearance. **(B)** Enlarged bilateral lateral ventricles are indicated by arrows. **(C)** Malmyelination or demyelination of white matter are indicated by arrows.

This research was approved by Dongguan People's Hospital (Dongguan, China) ethics committee. The family originated from the Guangdong province in mainland China. The proband was a boy aged 5 years and 8 months old, and his parents had a normal phenotype. There was no history of intellectual disability in the family, and consanguinity was denied. No complications were found during pregnancy. The patient was delivered at 40 weeks by Cesarean section due to suspected fetal macrosomia. Neonatal asphyxia was denied. The proband's current physical development was a weight of 16.5 kg [−3 standard deviation (SD)], a height of 107 cm (−2 SD) and head circumference of 43.5 cm (the size of a 6-month-old). Distinctive features included orbital hypertelorism, a flat nasal bridge, lower lip evagination and excess salivation (Figure 1A). All the milestones were markedly delayed, and the patient still could not walk or perform daily activities on his own. He could make sounds and had normal hearing, but could not speak. Recurrent respiratory infections leaded to inpatient treatment for times. His emotion was unstable and hard to comfort sometimes, but quiet in most time. He received an operation due to cryptorchidism in Dongguan People's hospital.

The patient's mother, a 34-year-old woman, gave birth to two children. The elder child was a healthy girl who was 12 years old, and the other was the proband. She became pregnant for the third time in 2020. Nuchal translucency of the fetus was 3.5 mm, crown-rump length was 65 mm, and a sonograph revealed that the kidneys were smaller than expected at 24 weeks' gestation. She received amniocentesis at 19 weeks due to the increased Nuchal translucency and abnormal child-bearing history. Both the karyotype and chromosomal microarray were normal. A novel hemizygous duplication mutation, NM_000489: c.189+1dupG, in the *ATRX* gene was detected by WES.

### Mutation Detection

Blood samples were collected from the patient and his parents, and an amniotic fluid sample was collected from the fetus through amniocentesis. Genomic DNA was extracted with a TIAMamp blood DNA kit (TIANGEN BIOTECH, Beijing, China) following the manufacturer's protocol.

The extracted DNA was fragmented randomly to 200–250 bp and was then purified using the magnetic particle method. The DNA fragments were ligated with adaptors and captured by probes of the IDT XGen Exome Research Panel (Integrated DNA Technologies, Coralville, IA, USA) targeting about 19,396 genes. The DNA libraries after enrichment and purification were sequenced on a NovaSeq 6000 sequencer according to the manufacturer's instructions (Illumina, San Diego, CA, USA). All reads were aligned to the reference human genome (UCSC hg19) by the Burrows-Wheeler Aligner (v.0.5.9-r16). After data annotation using the PriVar toolkit, the clinical significance of the variants was identified. The detected variants were validated with PCR, and the products were subjected to Sanger sequencing on a 3500XL Genetic Analyzer (Applied Biosystems, Foster City, CA, USA) according to the manufacturer's protocol.

### Bioinformatics Analysis

Mutations were classified as pathogenic, likely pathogenic, uncertain significance, likely benign and benign in accordance with the joint consensus recommendation of the American College of Medical Genetics and Genomics (8). Mutation Taster (http://www.mutationtaster.org/) was used to predict the genetic mutation effects, and the analysis of 5′ splice site score was estimated with MaxEntScan (http://hollywood.mit.edu/burgelab/maxent/Xmaxentscan_scoreseq.html). Changes in amino acid sequences caused by nucleotide mutations were estimated with Mutalyzer (http://www.mutalyzer.nl/). The three-dimensional structures of wild type and mutant ATRX protein were predicted with I-TASSER (https://zhanglab.ccmb.med.umich.edu/).

### RNA Analysis

Standard Trizol extraction was performed to extract mRNA from the peripheral blood of the patient, his mother and a healthy control. The HiScript II first Strand cDNA Synthesis Kit (Vazyme Biotech, Nanjing, China) was used to synthesize complementary DNA (cDNA). The total reaction system was 20 μl, and the total RNA template was 500 ng. SYBR Green (Vazyme Biotech, Nanjing, China)-based quantitative real-time PCR (qRT-PCR) was used to assess the expression of *ATRX* mRNA from the three samples. Differences in mRNA levels was assessed by the two-standard-curves method. Signal intensity from the *ATRX* gene was normalized to that of the *GAPDH* gene. The standard curve was the Ct value plotted against the log of the input mRNA concentration at five 10-fold serial dilutions. Primers were designed based on the sequence of the cDNA, covering the second to the fourth exons, with a product of 143 bp. Primers were as follows: Forward: 5′-AGAAACAAGTTCTCCTCCACGA-3′, Reverse: 5′-GTGACGATCCTGAAGACTTGG-3′. Target-gene expression was normalized to *GAPDH* expression. The qRT-PCR reaction was performed in triplicate, from which mean values and standard deviations were calculated based on the data obtained. mRNA expression levels were calculated with the 2^−Δ*ΔCT*^ method in the SYBR Green system. The Analysis of Variance was applied for statistical analysis, while a *p* < 0.05 was considered statistically significant. The product of the qRT-PCR analysis was subjected to Sanger sequencing.

## Results

### Clinical Data

No abnormalities were found in the karyotype and chromosomal microarray from peripheral blood from the patient. The results of routine blood examination, hemoglobin electrophoresis and sex hormone analysis are shown in Table 1. Epileptic discharges were observed in the electroencephalogram. A cranial Magnetic Resonance Imaging (MRI) showed multiple abnormal signal areas near the bilateral lateral ventricles, which were considered as malmyelination or demyelination of white matter. Enlarged bilateral lateral ventricles, widened and deepened sulci and fissures were found (Figures 1B,C).

**Table 1:** Biochemical parameters of the family under study.

**Parameters**	**Patient**	**Mother**	**Reference value**
Hb (g/L)	127	128	115–150
MCV (fl)	79.2v	88.5	82–100
MCH (pg)	27.3	29.5	27–35
Hb A (%)	96.1	97.4	96.5–97.5
Hb A_2_ (%)	2.6	2.8	2.5–3.5
Hb H(%)	negative	negative	negative
LH (mIU/ml)	0.48	/	1.24–8.62
PRL (mIU/l)	337.3	/	55.97–278.36
TSTO (nmol/l)	<0.35	/	6.07–27
GH (ng/ml)	0.572	/	0–1

*Hb, hemoglobin; MCV, mean corpuscular volume; MCH, mean corpuscular Hb; HbH, Hemoglobin H; LH, luteinizing hormone; PRL, prolactin; TSTO, testosterone; GH, growth hormone*.

### Identification of *ATRX* Mutations

**Figure 2:**
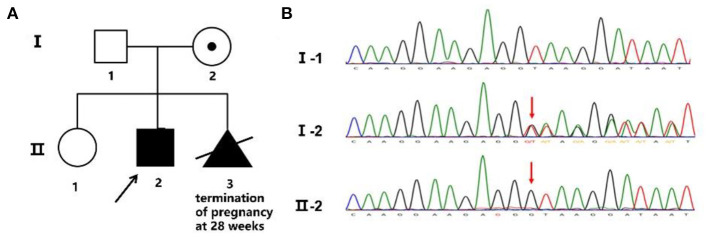
Family pedigree and Sanger sequencing. **(A)** Family pedigree. The proband's mother chose to terminate the third pregnancy at 28 weeks. **(B)** The novel mutation was validated by Sanger sequencing, and the red arrow shows the variant.

**Figure 3:**
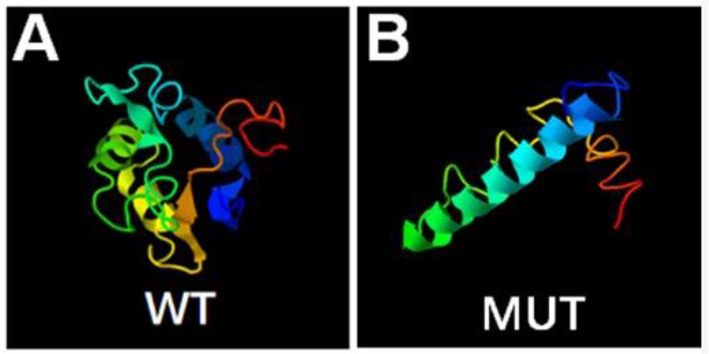
Bioinformatics analysis. Prediction of wild type **(A)** and mutant type **(B)** protein topology by I-TASSER.

A novel hemizygous duplication mutation in the *ATRX* gene in a splicing donor site between exons 3 and 4 was identified by WES from the patient and the fetus, NM_000489: c.189+1dupG. Sanger sequencing confirmed that the mutation was inherited from their mother, who carried the heterozygous mutation, while their father was not affected (Figures 2A,B). The novel mutation was classified as likely pathogenic, and it had not been previously reported. Mutation Taster software indicated that the duplication was likely to be disease causing. MaxEntScan showed a 5′ splice site score of −8.4 for the mutant type, markedly lower than 10.1 for the wild type, noting that the maximum 5′ score is 12.6. Mutalyzer predicted that a frameshift mutation was produced, which created a stop codon leading to a premature termination of translation at codon 196, manufacturing a truncated protein of 65 amino acids. I-TASSER predicted the length of the truncated protein, and the tertiary structure was changed significantly (Figures 3A,B).

### Functional Analysis

**Figure 4:**
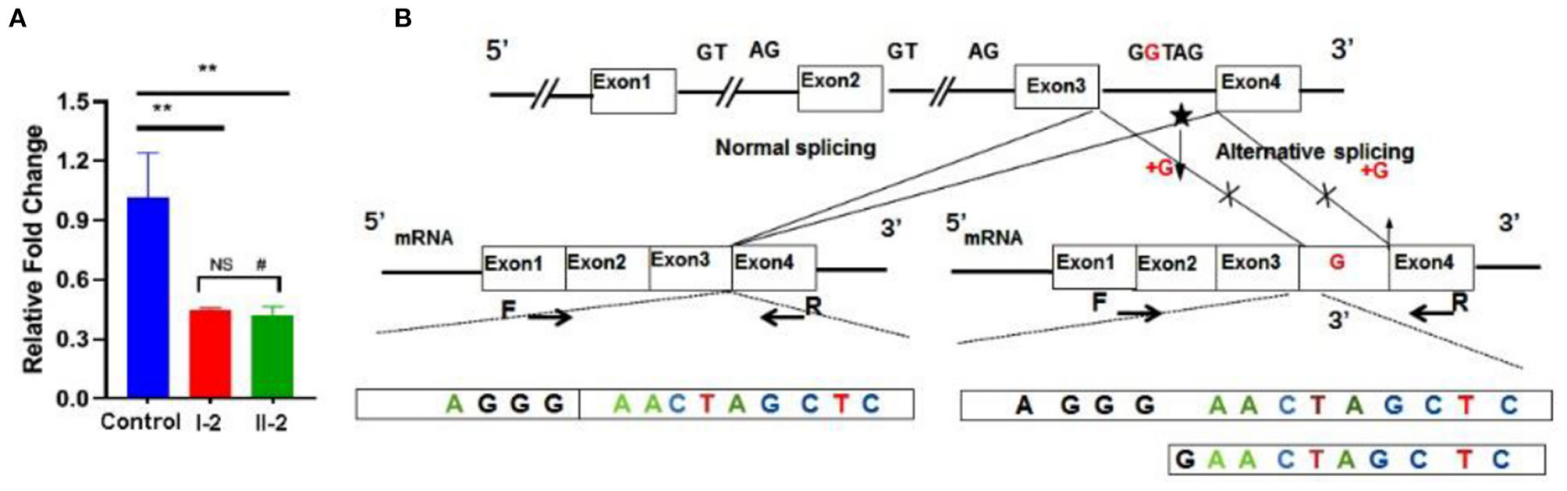
Functional studies. **(A)** mRNA expression levels were decreased significantly in the patient and his mother. **(B)** qRT-PCR product sequence chromatograms by Sanger sequencing showed a base insertion of G between exons 3 and 4 in the affected individual. **p < 0.05, ^#^non-significant.

Ideal amplification curves of the target gene and reference gene were obtained. Non-specific amplification products and primer-dimers were not found. The mRNA expression from both the patient and his mother were lower than that of the healthy control, and the difference was significant (*p* < 0.01). No statistically significant difference was found between the patient and his mother (*p* > 0.05; Figure 4A). Sanger sequencing of the qRT-PCR product indicated the insertion of a G between base 189 to 190 in the complementary DNA (Figure 4B).

## Discussion

ATR-X syndrome is a rare X-linked dominant disorder, first described by Gibbons et al. (1), with an estimated prevalence of 1–9/100,000 (7). Distinct from other X-linked dominant disorders, most cases of ATR-X syndrome occur in males. Carrier females seldom show clinical manifestations due to marked skewing of X-chromosome inactivation, with preferential inactivation of the X-chromosome carrying pathogenic variation being observed in heterozygous females (2). The *ATRX* gene, located on Xq13.3-21.1, encodes two transcripts, one of which encodes the full-length protein composed of 2,492 amino acids, while the other encodes a truncated protein generated by an alternative splicing event (7). The ATRX protein contains a zinc finger domain, which combines DNA and a helicase domain and functions in the transcription process to open double-stranded DNA (9). The ATRX protein belongs to the SWI/SNF2 family, which are ATP-dependent chromatin remodelers that play crucial roles in a broad range of biological functions such as transcriptional regulation, DNA repair and chromosome segregation (10). Mutations in this enzyme family lead to abnormal gene expression impacting cellular activities including DNA replication and repair, in addition to cell proliferation and differentiation, resulting in a broad range of neurodevelopmental disorders (11, 12). Moreover, functional studies have demonstrated that ATRX is a heterochromatin-interacting protein, in combination with chromatin-associated proteins such as MeCP2, HP1, and DAXX. Aberrant expression of the *ATRX* gene impinges on the interaction between the ATRX protein and proteins mentioned above, leading to intellectual disability (13–16). In this case, the proband suffered from severe developmental impairment, delayed milestones such as lifting, turning and sitting and could not stand independently at the age of 5. He was incapable of speech, pronouncing only a few sounds. Signs of seizure were found in the electroencephalogram, and abnormal signals were found in the MRI. Therefore, his phenotype was consistent with the clinical manifestations of ATR-X syndrome.

α-globin gene expression is down-regulated to different degrees when the *ATRX* gene is mutated (17, 18). This discrepancy results in varying extents of α-thalassemia, mainly reflected in different proportions of red cells containing hemoglobin H inclusion (0–30%) (19) and microcytic hypochromic anemia. This demonstrates that the regulation of α-globin by the *ATRX* gene is also influenced by other genes or proteins, but β-globin expression is not disturbed (18, 20). There is no significant correlation with the severity of intellectual disability and the degree of α-thalassemia (2). No abnormalities in hemoglobin electrophoresis were found in this pedigree, and no hemoglobin H inclusion was detected in the red blood cells. We found that the patient's mean corpuscular volume was <80 fl and mean corpuscular hemoglobin was <27 pg in multiple independent tests despite his hemoglobin being normal. This could be explained by the fact that the mutated *ATRX* gene down-regulated α-globin gene expression.

ATRX gene is an early “sex differentiation” gene (19). The mutant *ATRX* gene affects sex differentiation in early embryonic development, leading to hypospadias, undescended testes, a small penis, or even ambiguous genitalia (21). Tang and his colleagues found that male patients always had testicular tissue, concluding that the *ATRX* gene functioned in sex differentiation rather than sex determination. In this case, the patient was found to have bilateral cryptorchidism when he was born, and he received orthopedic surgery at 2 years old. The genital abnormality was very mild, but sex hormone levels were still low when he was more than 5 years old. It is still unknown whether androgen deficiency will affect adolescent development. We suggested that the patient's phenotype could result from a mutant gene. Going back to the fetus, a sonograph showed that the kidneys were smaller than expected at 24 weeks. However, we were unable to track the fetal genital development due to the termination of pregnancy at another hospital.

WES and Sanger sequencing validated a splicing insertion between exons 3 and 4 of the *ATRX* gene in this case (NM_000489: c.189+1dupG). The nucleotide sequence in the splicing region where the variation is located is highly conserved. The insertion of a G base gives rise to anomalous splicing, which was confirmed by the sequencing of the qRT-PCR product. RNA splicing is a key step in gene expression in eukaryotes (22, 23). Splicing defects result in whole or partial exon deletion, whole or partial intron retention or deletion of multiple exons, leading to premature termination of translation or loss of amino acids, and can even cause cancer (24, 25). A frameshift mutation was predicted by Mutalyzer software, and the mutation resulted in an early stop codon at the 65th amino acid, generating a truncated protein with a molecular weight of only 7–8 kD, while the normal ATRX protein has a molecular weight of 283 kD with 2,464 amino acids. The I-TASSER database predicted that the conformation and length of the truncated protein changed significantly, affecting the protein function. We hypothesize that loss of function of the mutant protein resulted in haploinsufficiency. Further functional verification is expected to identify the effect of the truncated protein on protein interactions.

It has been reported that *ATRX* is a dose sensitive gene in a previous study. Research showed that mice with a complete knockout of the *ATRX* gene died at day 9.5 (19). On the other hand, high embryonic lethality was observed in transgenic mice with overexpression of *ARTX*, and also craniofacial anomalies and defects in the organization of the cortex in survivors (25, 26). Mutated *ATRX* impacts *ATRX* mRNA expression to varying degrees. McDowell et al. carried out a Western blot assay with monoclonal antibodies against ATRX, revealing that the level of ATRX protein decreased remarkably in ATR-X syndrome patients, and was even undetected in some of them (27). However, there was no significant correlation between the degree of retardation and the level of ATRX protein (2). In this study, qRT-PCR showed that the level of mRNA in the patient and his mother were markedly lower than that in a healthy control, which is consistent with previous research. This result agreed with the occurrence of non-sense-mediated mRNA decay process, which is a conserved mRNA monitoring way that cells use to ensure the quality of transcripts and to fine-tune transcript abundance (28). Interestingly, no significant difference was found between the patient and his mother, who was a carrier. One interpretation of this result might be the phenomenon of skewed X-chromosome inactivation in X-linked intellectual disability diseases (29). Mary R et al. built the *ATRX*-mutant mouse model and carried out immunohistochemical analysis for ATRX expression. There were evidences to see independent tissue-specific effects in the expression analysis and skewed inactivation was more obviously in cerebral cortex (30). We then attempted to carry out functional studies on the protein. However, Western blot could not be carried out successfully due to the large molecular weight of the ATRX protein and the small molecular weight of the truncated protein, which might be degraded in the body, leading a failed experiment. Another limitation of this study should be mentioned is that the melting curve used in qPCR assay could not distinguish between the correctly spliced transcript and the abnormal one, where only one single nucleotide is inserted in the mature mRNA. Validations were performed through agarose gel electrophoresis with qRT-PCR product, but no abnormal bands were detected.

In summary, we describe a male aged 5 years and 6 months with ATR-X syndrome in a Han Chinese family. A novel mutation was detected by WES, which was shown to be pathogenic through functional analysis. Our research has expanded the spectrum of *ATRX* gene mutations, providing a basis for a precise diagnosis and genetic counseling.

## Data Availability Statement

The original contributions presented in the study are included in the article/supplementary material, further inquiries can be directed to the corresponding author/s.

## Ethics Statement

The studies involving human participants were reviewed and approved by Ethics Committee of Dongguan People's Hospital (Dongguan, China). Written informed consent to participate in this study was provided by the participants' legal guardian/next of kin. Written informed consent was obtained from the individual(s), and minor(s)' legal guardian/next of kin, for the publication of any potentially identifiable images or data included in this article.

## Author Contributions

SW: writing—original draft preparation. YZ: methodology. CX and JF: data curation. FY and FX: writing—review and editing. All authors contributed to the article and approved the submitted version.

## Funding

This work was funded as follows: (1) Clinical research nurturing program of Southern Medical University (LC2016PY021); (2) Special project on clinical research of Nanfang Hospital (2018CR039); (3) Natural Science Foundation of Guangdong Province (2021A1515011649); (4) Guangzhou Basic and Applied Basic Research Foundation (202102080103); (5) The key project of social science and technology development of Dongguan (2018507150011651); and (6) The key project of social science and technology development of Dongguan (201950715001208).

## Conflict of Interest

The authors declare that the research was conducted in the absence of any commercial or financial relationships that could be construed as a potential conflict of interest.

## Publisher's Note

All claims expressed in this article are solely those of the authors and do not necessarily represent those of their affiliated organizations, or those of the publisher, the editors and the reviewers. Any product that may be evaluated in this article, or claim that may be made by its manufacturer, is not guaranteed or endorsed by the publisher.
